# Fine root lignin content is well predictable with near-infrared spectroscopy

**DOI:** 10.1038/s41598-019-42837-z

**Published:** 2019-04-23

**Authors:** Oliver Elle, Ronny Richter, Michael Vohland, Alexandra Weigelt

**Affiliations:** 1 Systematic Botany and Functional Biodiversity, Institute of Biology, Leipzig University, Johannisallee 21-23, D-04103 Leipzig, Germany; 2 Geoinformatics and Remote Sensing, Institute for Geography, Leipzig University, Johannisallee 19a, D-04103 Leipzig, Germany; 3 German Centre for Integrative Biodiversity Research (iDiv) Halle-Jena-Leipzig, Deutscher Platz 5e, D-04103 Leipzig, Germany; 4 Macromolecular Chemistry and Paper Chemistry, Ernst-Berl-Institute, Technische Universität Darmstadt, Alarich-Weiss-Straße 8, D-64287 Darmstadt, Germany

**Keywords:** Biological techniques, Biodiversity

## Abstract

1. Root lignin is a key driver of root decomposition, which in turn is a fundamental component of the terrestrial carbon cycle and increasingly in the focus of ecologists and global climate change research. However, measuring lignin content is labor-intensive and therefore not well-suited to handle the large sample sizes of most ecological studies. To overcome this bottleneck, we explored the applicability of high-throughput near infrared spectroscopy (NIRS) measurements to predict fine root lignin content. 2. We measured fine root lignin content in 73 plots of a field biodiversity experiment containing a pool of 60 grassland species using the Acetylbromid (AcBr) method. To predict lignin content, we established NIRS calibration and prediction models based on partial least square regression (PLSR) resulting in moderate prediction accuracies (RPD = 1.96, R^2^ = 0.74, RMSE = 3.79). 3. In a second step, we combined PLSR with spectral variable selection. This considerably improved model performance (RPD = 2.67, R^2^ = 0.86, RMSE = 2.78) and enabled us to identify chemically meaningful wavelength regions for lignin prediction. 4. We identified 38 case studies in a literature survey and quantified median model performance parameters from these studies as a benchmark for our results. Our results show that the combination Acetylbromid extracted lignin and NIR spectroscopy is well suited for the rapid analysis of root lignin contents in herbaceous plant species even if the amount of sample is limited.

## Introduction

Soils are a major sink of terrestrial carbon (C)^1^ primarily via plant litter. Litter decomposition thus contributes a substantial amount of C to the fluxes in soils and from soil to atmosphere and represents an important link between plant productivity and C stocks in the soil^2^. For grassland ecosystems, up to 70% of plant biomass is stored in the roots^3,4^, producing litter with closer contact to soil particles and longer residence times in soils than other plant tissues^5^. Thus, especially in grasslands, root decomposition constitutes a major C source^2,6^. Root lignin is one important driver of root decomposition^7^ but lignin quantification is labor-intensive especially for large sample sizes as often used in ecological studies. Here, we present a method to predict fine root lignin content from high-throughput near infrared spectroscopy (NIRS) measurements to overcome this bottleneck.

Root decomposition is driven by three major processes: chemical litter composition, soil biota and soil abiotic environment^8,9^. In grasslands, root chemical composition and specifically lignin and the carbon to nitrogen ratio are the major drivers for root decomposition^10–12^. High contents of holocellulose and nitrogen are positively correlated whereas a high lignin content is negatively correlated with root decomposition^13–16^. Lignin is the second most abundant biopolymer on Earth^17^ and represents a class of aromatic macromolecules characterized by complex, three-dimensional structures, which increase the stability of cell walls^18^. Lignification of plant tissues therefore increases their recalcitrance against chemical, biotic and abiotic degradation.

There is a multitude of methods for lignin determination^19^. The most common method of gravimetric determination of an acid insoluble residue (Klason lignin^20^) or the related acid detergent lignin method (van Soest method^21^) both require sample volumes of 500 mg to 5 g for accurate results^22^. For smaller sample volumes such as herbaceous samples or fine roots (usually ≤10 mg), the acetyl bromide (AcBr) extraction offers an alternative approach to make lignin soluble for quantitative UV absorption measurement^23–25^. However, both methods are labor-intensive and not well-suited to handle large numbers of samples as commonly examined in ecological studies.

Ecological models, by their very nature, have to consider a high biological variation between species, taxa, sites or experimental treatments. Near infrared spectroscopy (NIRS) is a fast and economic approach to predict chemical compounds in such large sample sizes. NIRS has been used successfully for a variety of compounds and materials i.e. chemical and biological properties of soils^26^, non-structural carbohydrates in plant tissues^27^, cellulose in paper based materials^28^ as well as for lignin in plant materials^29^. NIRS is based on the absorption of photon energy ranging from 800 nm–2500 nm and the excitation of molecular overtone and combined vibrations from mainly hydrogen containing chemical groups^30,31^. NIR spectra of lignocellulosic materials reveal complex absorption patterns due to the high chemical diversity of plant material including that of lignin^30^.

Chemometric methods are essential to model the relationship between NIR spectra and measured analytical constituents^30^. The most widely used chemometric method is partial least squares regression (PLSR)^32,33^ which uses the complete NIR spectra to define latent variables as combinations of the original spectral variables. However, this regression technique does not eliminate the problem of uninformative or even noisy spectral data that may affect the modelling approach. Therefore, combining methods which select the most informative spectral variables with the PLS regression often provides models with a greater predictive power^34^. There are several comprehensive overviews of such variable selection techniques for PLS models^34–36^. In principle, these methods can be subdivided into two groups: (1) methods considering only separate effects of the introduced statistical features and (2) methods considering the interaction of variables in the search space (which is often linked to a more exhaustive search compared to the approaches of the first group)^36^. The competitive adapted reweighted sampling (CARS) algorithm^37^ belongs to the first group of methods. It often provides more accurate estimates than full spectrum-PLSR^38^. CARS algorithm was successfully benchmarked against other state of the art variable selection techniques in regression tasks such as genetic algorithm (GA)^39^, successive projections algorithm (SPA) and iteratively retaining informative variables (IRIV)^38^. CARS algorithm works in a fast and computationally efficient way even for a great number of spectral bands^36,38^. Furthermore, CARS-PLS-DA models were significantly superior in classifying hyperspectral images as compared to non-parametric support vector machines (SVM) and random forest (RF)^40^. Moreover, the identity of wavelengths selected with CARS algorithm in a final model might inform the physical interpretation of results due to the link to specific chemical groups or bindings^37,41,42^. In addition to spectral variable selection, the pre-processing of NIR spectra is an integral part of chemometric modeling^43^. Rinnan *et al*.^43^ systematically compared a wide range of different pre-processing methods for moisture and sugar contents in marzipan samples. According to their results, the choice of the best pre-processing technique could improve model prediction up to 25% relative to unprocessed NIR spectra.

To put our results in a wider methodological and statistical perspective we performed a literature survey for studies predicting lignin content via spectroscopic measurements. We listed studies (1) measuring chemical content of lignin and using spectroscopy on plant tissue samples, (2) reporting the set of species used for lignin extraction, (3) using PLSR or other multivariate calibration methods to predict lignin contents spectrally and (4) validating the model on independent test data. We identified 30 relevant studies, which predicted lignin content via spectroscopic measurement following our four prerequisites. These studies reveal that NIRS has been used to predict Klason lignin or comparable methods of lignin analysis in wood^44,45^ or leaves^46,47^ of mainly silvicultural species such as pine, birch or eucalyptus. In addition, a number of studies measured lignin in agriculturally relevant species such as rice, corn, elephant grass or bamboo^29,48–50^. However, we did not find any study evaluating the prediction of acetyl bromide extracted lignin with NIRS nor a study trying to predict lignin in root material.

Therefore, the goals of this study are (i) to test, for the first time, if NIR based models can predict AcBr extracted fine root lignin content, and (ii) to explore whether it is possible to predict root lignin content in mixtures of up to 60 different herbaceous grassland species. Our chemometric approach to link lignin content to NIR spectra uses two steps. First, we evaluate the benefits of different spectral pre-processing techniques and second we compare the performance of model prediction using either full spectra or spectral variable selection (with the CARS algorithm). As plant material, we used biomass of fine roots (i.e. <2 mm in diameter) sampled from different plant communities of the Jena Experiment, a large grassland biodiversity experiment in Thuringia, Germany.

## Results

### AcBr based fine root lignin content

We found a lignin content of 20.52 ± 7.7% (mean ± SD) in the fine roots of mixed grassland communities of the Jena Experiment. The 74 samples spanned a species gradient of 1–16 species from a pool of 61 species with a minimum lignin content of 7.7% and a maximum of 42.8%.

The RSEL of ±3.61% is within the range of other studies reporting RSEL for AcBr based lignin determinations^51,52^.

### Spectral Pre-processing

**Figure 1:**
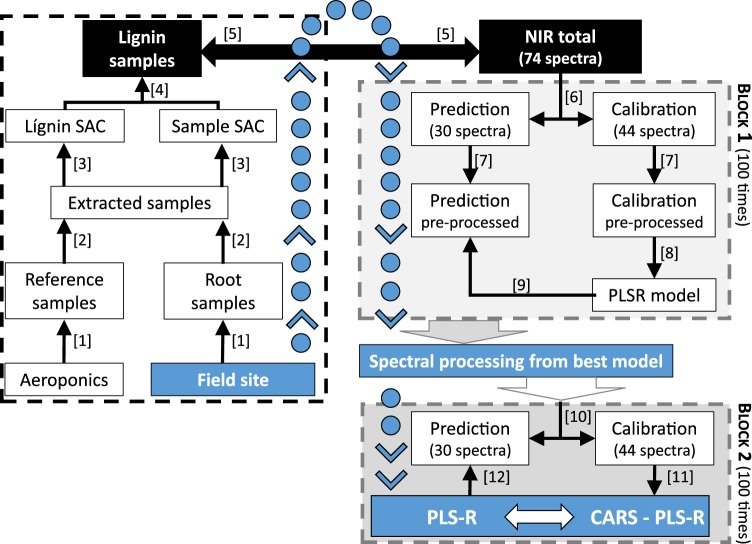
Schematic depiction of the workflow of chemical analysis (left box with steps 1–4 indicated in square brackets), spectral analysis (outlined in black boxes and step 5) and statistical analysis. The latter is subdivided in spectral pre-processing (block 1 with steps 6–9) and final analysis (block 2 with steps 10–12). See text for further details.

We evaluated the effect of six different methods of spectral pre-processing on the accuracies of full spectrum PLSR in 100 repeated independent model validations (Fig. 1). We based our model evaluation on root mean square error of prediction (RMSEP) and residual predictive deviation (RPD) (Table 1). Standard normal variate (SNV) pre-processing achieved the overall highest model accuracies with highest RPD and lowest RMSEP values. This pre-processing was therefore selected for further modelling in block II (Fig. 1). Overall accuracies were lowest for multiplicative scatter correction (MSC) yet the high BIAS and RMSEP values indicate a strong offset between predicted and observed lignin content. Irrespective of spectral pre-processing, all full spectrum PLSR-models showed RPD values below 2 and were therefore not promising to accurately predict lignin in quantitative analyses.

**Table 1:** Accuracies for different pre-processing methods achieved in model validation (mean and standard deviation from 100 runs).

Method	nLV	RPD	R_P_^2^	RMSEP [%]	SEP [%]	BIAS [%]
Raw	8.17 ± 0.75	1.83 ± 0.32	0.71 ± 0.08	4.11 ± 0.62	4.05 ± 0.59	−0.43 ± 0.95
B.als	6.62 ± 1.48	1.73 ± 0.27	0.69 ± 0.07	4.34 ± 0.64	4.27 ± 0.59	−0.44 ± 1.05
B.irls	7.96 ± 1.40	1.69 ± 0.30	0.68 ± 0.09	4.47 ± 0.69	4.40 ± 0.65	−0.49 ± 1.04
MSC	7.34 ± 1.61	1.95 ± 0.37	0.74 ± 0.09	11.64 ± 8.74	3.84 ± 0.64	−1.83 ± 13.97
**SNV**	**7.37 ± 1.28**	**1.96 ± 0.35**	**0.74 ± 0.08**	**3.83 ± 0.57**	**3.79 ± 0.56**	**−0.22 ± 0.86**
D1	7.95 ± 2.08	1.84 ± 0.39	0.71 ± 0.10	4.14 ± 0.73	4.09 ± 0.70	−0.35 ± 0.98
D2	5.04 ± 2.31	1.56 ± 0.17	0.58 ± 0.09	4.77 ± 0.64	4.71 ± 0.59	−0.02 ± 1.18

Given are measures of accuracies as number of latent variables used in PLSR (nLV), residual predictive deviation (RPD), measure of determination between predicted and observed lignin contents (R_p_²), root mean squared error of prediction (RMSEP), standard error of prediction (SEP) and deviation from the line of equality of linear regression between predicted and observed values (BIAS). We used raw data (Raw) and six different pre-processing methods: asymmetric least squares baseline offset correction (B.als), iterative restricted least squares baseline offset correction (B.irls), multiplicative scatter correction (MSC), standard normal variate (SNV), first derivative (D1) and second derivative (D2). The best model is highlighted in bold.

### Variable selection

**Figure 2:**
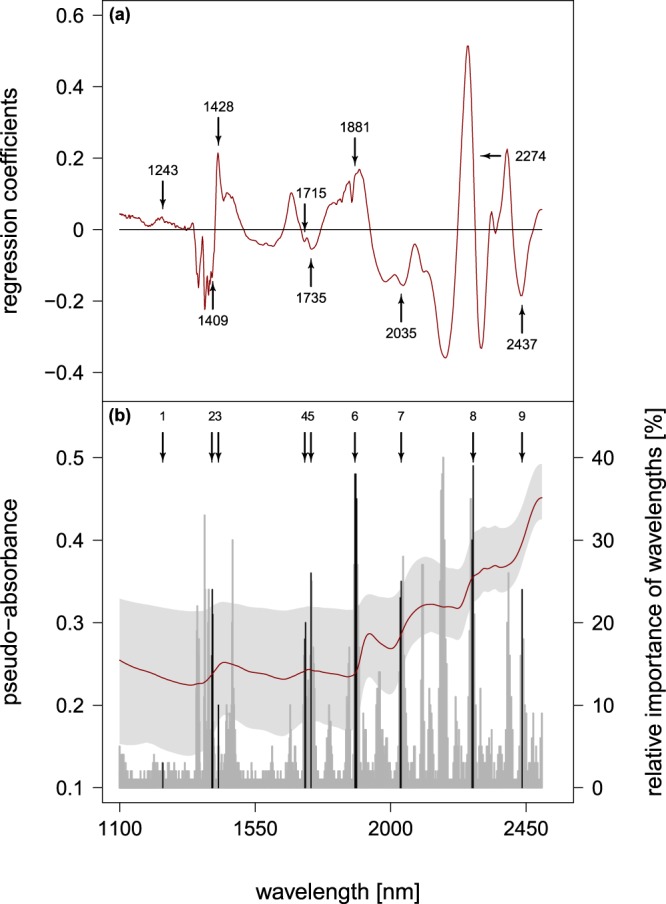
(**a**) Regression coefficients from full spectrum PLS (red line) with the selected wavelengths from the best CARS-PLS model as overlay (black arrows with wavelength indicated). (**b**) Mean absorbance spectrum of fine roots (red line) and its range (light grey) together with the relative importance (in %) of wavelength selection during 100 runs (dark grey bars) and the most important variables from the best model (black bars). Black arrows and number refer to the wavelength clusters indicated by the most important selected variables.

The variable selection procedure based on CARS-PLSR significantly improved the accuracies in independent model validation to RPD values above 2.5 (Table 2). Starting from the full spectrum, the RMSE steadily declined during the variable selection process such that the remaining wavelengths are more likely to allow better predictions. Moreover, CARS-PLSR identified wavelength regions, which code for the most important information within the regression procedure and thus potentially facilitate a mechanistic understanding of model results. The best CARS-PLSR model used 16 wavelengths, partly adjacent so that they formed nine clusters over the whole spectral region (as indicated by arrows and black bars in Fig. 2b). All wavelengths with PLS regression coefficients >0 are positively correlated with lignin content, whereas wavelengths with regression coefficients <0 indicate a negative relationship with lignin content and are most likely linkable to other chemical compounds (e.g. cellulose and polyoses). The selected 9 wavelength clusters from the best model can be assigned to local extrema of the regression coefficients obtained through PLSR modeling (Fig. 2a). The relative importance of selected wavelengths in the CARS-PLSR increases from lower to higher wavelengths with local maxima around 1428 nm, 1881 nm and 2274 nm (Fig. 2a) but increasing importance for the prediction of lignin content above the cluster of 1881 nm (Fig. 2b). However, our results also imply that wavelengths below 1881 nm likely have strong supplementary qualities since prediction accuracies strongly decreased if single wavelengths within that region were excluded from our model. Our best model indicated four wavelength clusters, which were positively correlated with lignin content (clusters 1, 3, 6 and 8 corresponding to wavelength 1243 nm, 1428 nm, 1881 nm and 2274 nm, respectively). At the same time, we found five clusters which were negatively correlated with lignin content (clusters 2, 4, 5, 7, 9 corresponding to wavelength 1409 nm, 1715 nm, 1735 nm, 2035 nm and 2437 nm, respectively).

**Table 2:** Comparison of accuracies (mean ± SD) achieved for full spectrum PLSR versus best model from variable selection using CARS-PLS resulting from 100 repetitions for the calibration and validation.

Models (S = 100)	WL-Selection	Full spectrum PLSR	CARS-PLSR
Calibration	Variables	662	16
	nLV	7.37 ± 1.28	6.99 ± 0.58
	RMSE [%]	3.58 ± 0.54	2.75 ± 0.30
Validation	RPD	1.96 ± 0.35	2.67 ± 0.46
	R_P_^2^	0.74 ± 0.08	0.86 ± 0.05
	RMSEP [%]	3.83 ± 0.57	2.82 ± 0.40
	SEP [%]	3.79 ± 0.56	2.78 ± 0.40
	BIAS	−0.22 ± 0.86	−0.19 ± 0.65

Abbreviations are as given in Table 1. In addition we use root mean square error of calibration (RMSE).

**Figure 3:**
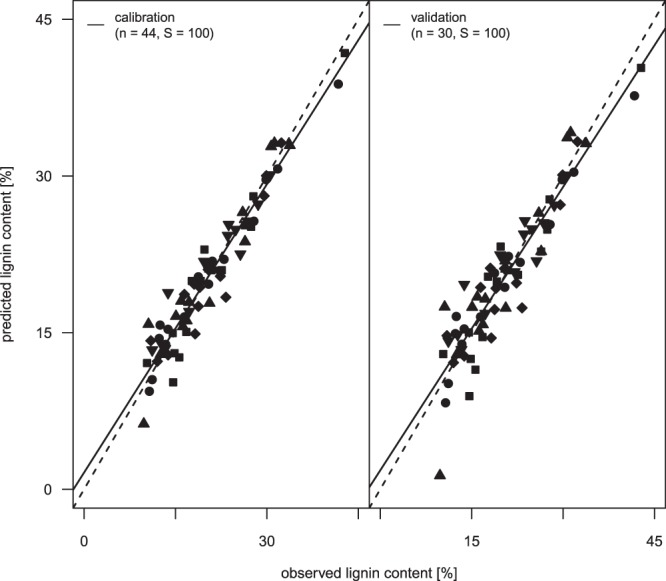
Predicted versus observed lignin contents (mean over 100 runs) from model calibration (left) and model validation (right) given as linear regression (dashed line) against the 1:1 line (solid line). Symbols represent the gradient of species richness (squares = 1, circles = 2, triangle = 4, rhombus = 8, triangle (upside down) = 16). n indicates the number of samples used in calibration and validation.

The gradient in species richness had no significant negative effect on model residuals in calibration or in validation (Fig. 3).

### Literature survey

Our literature survey identified 30 papers, which predicted lignin content (extracted either based on Klason or the van Soest method) in plant tissue via spectroscopic measurements. These papers reported details on 38 case studies either as different species within one paper or different tissue (e.g. stem and leaves) from the same species (see Supplementary Table S1 for full details of individual studies). The literature survey revealed three critical issues: First, 58% of the case studies analyzed lignin content in woody tissue, where a higher lignin content results in a more pronounced spectral signal. Second, 78% of the case studies were limited to one species and only 8% had more than five species. This minimizes the complexity of spectral signals reported in literature, as interspecific chemical variability is likely to be higher than intraspecific variability in chemical compounds^53,54^. Third, the minimum amount of sample was 100 mg and the median amount 300 mg, which is one order of magnitude higher compared to this study (10 mg).

Table 3 summarizes the results of this literature survey as quantile ranges of common measures of accuracy and predictive power (RMSEP, SEP, R^2^ and RPD). The median accuracy over these 38 case studies indicates an acceptable quantitative prediction of lignin content in plant tissue with a RPD of 2.45 and R² of 0.83 in validation. In comparison, our best model had a better quantitative prediction (RPD of 2.67) and a higher predictive R² of 0.86.

**Table 3:** Summary statistics of a literature survey of studies predicting lignin contents from different plant tissues using spectroscopic methods.

	Species^38^	Lignin [mg]^36^	Val/Cal^35^	RPD^28^	${{\bf{R}}}_{{\bf{P}}}^{{\bf{2}}}$ ^33^	RMSEP [%]^19^	SEP [%]^21^
Min	1	100	0.12	0.5	0.05	0.19	0.28
Q25	1	275	0.29	1.61	0.61	0.68	0.65
Q75	1	500	0.50	3.56	0.90	1.88	2.27
Max	32	2500	3.55	9.5	0.99	3.48	5.00
**Med**	**1**	**300**	**0.38**	**2.45**	**0.83**	**1.03**	**0.87**
**This study**	**61**	**10**	**0.68**	**2.67**	**0.86**	**2.82**	**2.78**

Given are minimum, maximum and interquartile ranges of the number of species examined per study (Species), sample volume used for chemical lignin extraction (Lignin) and proportion of samples used for model validation and calibration (Val/Cal). Additional abbreviations as in Table 1. Superscript numbers indicate the number of individual datasets that entered the analysis. See Table S1 for details on individual studies.

## Discussion

Our study demonstrates that fine root lignin content is well predictable using near infrared spectroscopy. This is true for field samples from monocultures up to 16 species mixtures assembled from a pool of 60 different herbaceous grassland species. Further, we find a higher accuracy in model prediction when combining PLSR with CARS variable selection (RMSEP = 2.82) as compared to models using the full spectrum PLSR (RMSEP = 3.83). All measures of accuracy for lignin prediction in our study were in the range of results from 38 relevant case studies extracted from a literature survey, despite much lower sample volumes and higher sample variation in our study. Thus, we could show that the combination of Acetylbromid extracted lignin and FT-NIR spectroscopy is well suited for the rapid analysis of root lignin contents of herbaceous plant species even if the amount of sample is limited.

### Fine root lignin content and range

The range of lignin content based on Acetylbromid extraction in our fine root samples (10–43%) exceeds that typically reported in the literature. However, most studies report lignin content in aboveground biomass based on Klason lignin extraction for woody gymnosperms (25–33%)^55^, woody angiosperms (20–25%)^54^ or herbaceous angiosperms (3–19%)^51,56^. There are three potential reasons for the discrepancy in lignin ranges between our study and literature values. First, the extraction methods might produce different results. Though Acetylbromid is better correlated to Klason lignin than other extraction methods^51^, there is an offset between both methods for herbaceous species^56^, which might account for part of the range differences we found. Second, roots often have higher lignin content than shoots^57^. The limited data available on lignin content of grass species fine roots suggest a mean of 19.6% ± 1.7%, which is only slightly lower than our 20.5% ± 7.7% for mixed herb species^7,57^. Third, our samples most likely contained a larger proportion of partially degraded fine root debris for which lignin content is known to be higher^47^. The field samples of mixed species were harder to sort for dead roots given the different color and texture of roots of different species. This problem might have been less pronounced for the data on individual species root lignin content published so far.

### Spectral pre-processing

We tested six different types of spectral pre-processing and raw spectra to determine the most adequate method for our sample type. We found that standard normal variate (SNV) and multiplicative scatter correction (MSC) obtained the highest prediction accuracies according to residual predictive deviation (RPD) and R² in validation. Both types of spectral pretreatments have been specifically designed for correcting NIR spectra of scattered radiation^58^ and have been widely applied in NIR spectroscopy^32^. Yet, Yet, error terms of prediction (RMSEP and BIAS) were higher in MSC compared to SNV in MSC compared to SNV. This difference can be traced back to the fact that we performed an independent spectral pre-processing for the validation and calibration set. Since MSC relies mathematically on the mean spectrum of the entire dataset^59^, any pre-processing results diverge for calibration and validation datasets. Thus, prediction accuracies widely change for individual random draws of validation and calibration data. In contrast, SNV acts on the individual spectrum only, which makes it less dependable on pre-processing results^60^. In addition, our results reveal that first and second derivative standardization, which have been found valuable in previous studies^45,46,61^ did not result in enhanced accuracies compared to using unprocessed spectra (raw spectra). This might indicate relatively minor contributions of spectral signal distortion in the data. While it remains difficult to a priori select the best pre-processing method^43^, the case of MSC revealed that it is possible to choose an incapable one.

### Selection of key wavelength-clusters based on CARS-PLS

We compared model accuracies between partial least square regression (PLSR) models and the combination of PLSR models with a predictor selection algorithm (competitive adaptive reweighted sampling, CARS). Our results indicate a significant increase in model validation accuracies through variable selection with CARS for all measures of model accuracy. Most likely, CARS predictor variable selection reduces the noise caused by non-informative variables for each individual model run. These findings are in line with other studies combining PLS based models with the CARS framework for variable selection^27,37,38,40^, also reporting an enhanced model performance compared to using the full set of predictors from PLSR models alone.

In addition to increased model accuracies, the selection of 9 clusters of wavelengths from the best model might also enhance our mechanistic understanding of spectral lignin prediction. The finding that wavelength cluster 1, 3, 6 and 8 (1243 nm, 1428 nm, 1881 nm and 2274 nm) are positively correlated with lignin content (indicated by positive regression coefficients in PLS) is in line with previous studies where these wavelengths seemed indicative of lignin or lignified structures. Li *et al*.^50^ also found the wavelength at 1243 nm to be relevant for predicting lignin content, but in contrast to our study, identified a larger set of important wavelengths (20), many of which located in the spectral region between 1450 nm and 1700 nm. The absorption at 1428 nm is often related to the first overtone of phenolic O-H stretching in lignin^62–64^. The cluster at 1881 nm is ascribed to C-H^62^ and O-H stretching and deformation^65^ and is associated with lignin in general^62^. The cluster around 2270 nm may also arise from O-H stretching overtones in lignin^63,64^. Thus, all selected positively correlated wavelength clusters can be directly linked to lignin or chemical structures related to lignin, which further strengthens the applicability of near-infrared prediction of fine root lignin content.

Similarly, the wavelength clusters 2, 4, 5, 7 and 9 (1409 nm, 1715 nm, 1735 nm, 2035 nm and 2437 nm) which were negatively correlated with lignin content, have previously been linked to chemical groups with some ecological counterpart or trade-off towards fine root lignin content.

During lignification, the protein containing, holocellulosic fiber scaffolding of the compound middle lamella and the secondary cell wall are incrusted with lignin^66^. This process increases the mechanical stability of lignified tissue against high compression and capillary forces. In addition, lignification enhances the recalcitrance of plant tissue against the alkaline soil solution, root exudates and exoenzymes from bacterial and fungal communities^5,67^. According to Schwanninger *et al*.^30^ wavelengths around 1715 nm and 1735 nm result from overtone C-H stretching vibrations in polyoses and cellulose. The wavelength cluster around 2035 nm could be connected to amide-1 stretching in peptides and proteins^63,64,68^ or similarly to C=O stretching vibration from acetyl groups in polyoses^50^. Bands at 2437 nm probably indicate α-helical protein structures^63^. Thus, the wavelength clusters 4, 5 and 7, which are negatively correlated to root lignin in our study, are indicative of cellulose, polyoses and proteins, indicating a trade-off well known from literature^69^. This trade-off may be explained by a substrate competition for the biosynthesis of cellulose and protein in a living cell, versus biosynthesis of lignin and the subsequent death of the cell^70^.

A potentially competing chemical group, providing similar functionality to the cell as lignin, are suberins. Suberins and associated waxes are important hydrocarbon-containing biopolymers that also increase the mechanical stability of plant tissue yet without impairing the water absorption capacity and plasticity of the root tissue. Our wavelength cluster 2 (around 1409 nm) has been connected to C-H stretching and deformation in aliphatic hydrocarbons^64,65^ and hydrocarbon-containing plant waxes^68^ as well as to O-H deformations in extractable alcohols^30,64,65^. Again, there might be a trade-off in allocating carbon to enhance tissue stability via either lignin or suberin and associated waxes. Suberins occur in the secondary walls of endodermal and hypodermal cells of primary roots, while the incorporation of waxes into the suberin polymer is limited to root tissues with secondary growth^71^. Yet, monocotyledonous roots, such as grasses, have no secondary growth and should invest into higher lignin concentration at the expanse of wax contents. Indeed, Zeier and Schreiber^72,73^ could show an inverse relationship between endodermal lignin and suberin content in five monocotyledonous species. In addition, Chen *et al*.^11^ could show that grass containing plots from the same experiment had significantly higher lignin contents and lower nitrogen contents in fine roots than legume containing plots.

Overall, our results show that wavelength assignments in NIRS have the potential to reveal insightful information regarding the underlying chemistry. Wavelengths with high explanatory power in the regression model provide us with background knowledge on the chemical groups or bindings, which absorb at distinct wavelengths^74^. However, this is not a trivial task in complex lignocellulosic materials^30^ since exact band positions depend on different equilibrium moisture contents^75^ and thus intrinsically on the chemical composition of constituents.

### Model design and best model outcome

The aim of our study was to develop a model to predict fine root lignin content, which is well applicable to a larger range of herbaceous species. For this reason, we put a strong focus on evaluating the predictive power of our models. Consequently, we assigned a much larger than usual proportion of samples to model validation. In addition, we used repeated outer validation instead of single cross-validation, as this allows for a more realistic determination of uncertainty when predicting unknown samples^76^. Both procedures increase the reliability of our models yet at the cost of enhanced error terms especially given the overall limited number of samples.

Despite this conservative procedure our best model has a better quantitative prediction (higher RPD value of 2.67) and a higher predictive R² of 0.86 compared to the median of 38 case studies extracted from our literature survey (RPD = 2.45, R² = 0.83). In contrast, our measures of error of prediction (RMSE and SEP) are higher than the 75% quantile range of literature studies. This discrepancy is due to the fact that our data set comprised a much larger sample variability due to the higher number of species. We are aware of only one other study^47^ with comparable sample complexity using leaves, leaf litter and organic residue of 31 tree species to predict lignin content via near infrared spectroscopy (SEP = 5, R² = 0.76 and RPD = 2.1). Similar results (RMSEP = 5.5 and R² = 0.5) were achieved by Kelly *et al*.^77^ when predicting lignin content for 14 agricultural species used for fiber production, indicating that in fact sample heterogeneity might well determine the error of model prediction for lignin. This phenomenon is well known for model prediction via spectral analysis for biological applications^78^. However, Petisco *et al*.^46^ found that lignin content in tree leafs from 17 species was well predictable (SEP < 1), but in that study lignin content was spanning a smaller range and standard deviation then realized in our study. Overall, our results reveal that root lignin in a large number of herbaceous species is at least as well predictable as the median of studies predicting lignin content in one or few mostly woody or crop species.

## Conclusion

Our study explored - for the first time - the applicability of NIR spectroscopy to determine AcBr extracted root lignin content in mixtures of up to 60 grassland species. We used CARS-PLS to select the most relevant wavelengths for root lignin prediction and concurrently increased prediction accuracy compared to PLS models based on full spectrum information. Despite the large number of species and the limited amount of sample available in our study, we found higher prediction accuracies than the median of 38 case studies extracted from a literature survey. Thus, we conclude that predicting AcBr extracted root lignin from NIRS spectroscopy shows great potential to overcome the limitation of large sample sizes as commonly examined in ecological studies.

## Material and Methods

### Sampling

The Jena Experiment is a biodiversity experiment studying the effects of plant species and functional group richness on ecosystem functions. 60 mesophilic central European grassland species were classified into four functional groups: grasses (16 species), legumes (12 species), small herbs (12 species) and tall herbs (20 species). Detailed information about the design and main results of 15 years Jena Experiment is given in Weisser *et al*.^79^. For the current study, we collected roots of plant communities from 76 experimental plots, spanning the existing gradient of species richness (1, 2, 4, 8, 16) and functional group richness (1, 2, 3, 4). Community root collection is explained in detail in Chen *et al*.^11^. In brief, we excavated soil volumes with a surface area ranging from 20 × 10 cm to 40 × 15 cm and a depth of 20 cm depending on the spatial extent of the respective root biomass. Roots were soaked in tap water prior to washing them over a 630 µm-mesh sieve and removing coarse roots (>2 mm), debris and highly decomposed roots before drying (65 °C). Three samples did not contain enough fine root material and had to be excluded from further analysis, leaving 73 samples for this study. In addition, we included one sample of standardized *Lolium perenne* L. roots. We used this as a reference sample of young and fully clean grass roots contrasting our field root samples with low diameter but potentially higher age (and lignification) as well as partly decaying roots or soil residue. *L. perenne* plants were cultivated in aeroponics in the greenhouse of the Botanical Garden of Leipzig University over 4 months. Newly grown roots and shoots were cut to 3 cm length every 4 weeks and roots were dried (65 °C) and stored. Dry *L. perenne* roots from all harvests were shredded and thoroughly mixed before grinding multiple smaller portions of the large sample volume with a vibratory ball mill (MM 400, Retsch Technology GmbH, Germany). After grinding, the powder was again carefully mixed and oven dried again (70 °C, 48 hours). Our overall sample number was therefore 74 (73 field samples plus *L. perenne*). Moreover, we used homogenized root biomass as internal reference standard to test replicability and recovery rates of chemical analysis. This reference sample was comprised of a mixed field community root sample bulked from the 50 largest field samples described above. This sample contained a large variety of grassland species and was extracted and measured with each analytical run.

### Chemical analysis

The left box of Fig. 1 depicts the chemical analysis where individual steps are indicated with numbers in square brackets. We refer to these numbers in the following text. Dried root samples were ground with a vibratory ball mill (MM 400, Retsch Technology GmbH, Germany) and oven dried again (70 °C, 48 hours) before further processing ([1], Fig. 1). For liquid-solid pre-extraction we extracted up to 50 mg of sample with 12 mL solvent (acetone: ethanol: water; 5:3:2 volume) at 70 °C for 150 minutes, turning the tubes regularly. The extracted samples were centrifuged and washed three times before drying (70 °C, 48 hours). For lignin extraction, we used the acetyl bromide (AcBr) extraction as described by Iiyama & Wallis^24^, but avoided to use 70% perchloric acid that causes the formation of hydrobromic acid and unwanted acid catalyzed, chromophor-forming oxidation of polysaccharides [2]. In brief, we extracted 10 mg sample with 5 mL 25% (vol:vol) solution of AcBr in glacial acetic acid and heated the vials in an oil bath (70 °C, 60 min) with regular shaking to promote sample digestion. We chilled samples on ice (15 min), equilibrated to room temperature (30 min) and centrifuged. To mask strongly absorbing polybromide anions, 1 mL of the supernatant was diluted in 1 mL of 2 N NaOH and 8 mL glacial acetic acid. We included microcrystalline cellulose (Sigma-Aldrich, USA) as control^80^. Finally, we measured 3 mL of the sample solution at 280 nm in a spectrophotometer (Jasco V730, Jasco Labor- u. Datentechnik GmbH, Germany) to determine the specific absorption coefficients (SAC) [3]:

1 $$SAC=\frac{(O{D}_{S}-O{D}_{B})\ast F}{{W}_{d}}\ast ml\ast c{m}^{-1}\ast m{g}^{-1}$$

where OD_S_ = optical density of the sample, OD_B_ = optical density of the blank, W_d_ = weight of the sample and F [mL mg^− 1^] = dilution factor (=50) and d [cm] = diameter of the quartz cuvette. We calculated the SAC per sample as mean over all replicates per sample (2 ≤ n ≤ 4). Each replicate of a sample was measured on a different day.

To translate SAC into lignin content we used the reference sample from mixed field roots described above. Two subsamples individually underwent the same procedure as described above. In addition, we purified and isolated these reference samples as described in detail by Fukushima & Dehority^81^. For the calibration curve we diluted 10–750 µL extracted lignin aliquots in 8 mL masking solution, made up to 10 mL with blank solution (25% AcBr in acetic acid) and measured at 280 nm as detailed above. The lignin content (L) of all samples was calculated using regression eq. (2) [4].

2 $$L=\frac{(SAC-0.05)\ast 100}{13.06}\ast {\rm{ \% }}$$

### Spectral measurements

The ground and dried root samples were measured between 12489 cm^−1^ − 3594 cm^−1^ (800 nm -2782 nm) with 8 cm^−1^ spectral resolution in transmission (Multi-Purpose FT-NIR-Analyzer, Bruker Cooperation, USA). Transmission (T) was converted to absorbance via log_10_(1/T). Each sample was measured 5 times; spectra were subsequently averaged. Samples were shaken between each replicate to ensure a spectral recording of the sample-inherent variability [5].

### Statistical analysis

We used partial least squares regression (PLSR) to relate measured spectra of 74 collected samples (each with a total of 662 spectral bands as predictor variables) to the measured lignin contents of these samples (response variable) [5]. We used PLSR as the most widespread multivariate calibration method. PLSR is capable of handling a high degree of collinearity in the predictor variables^82^ as well as a small number of response samples in relation to the number of predictors^33^. In addition, we combined full spectrum-PLSR with the CARS algorithm, introduced and described in detail by Li *et al*.^37^. In short, CARS, aims at selecting key wavelengths in a computationally efficient way. The selection procedure in CARS is largely based on the PLS regression coefficients and consists of two major steps. In the first step an exponential decreasing function (EDF) defines the number of selected spectral variables. As a result, the number of kept variables decreases exponentially from one sampling run to the next and wavelengths linked to small absolute PLS regression coefficients are removed before the second step in each sampling run. This second step, also referred to as adaptive reweighted sampling (ARS), uses the absolute regression coefficients to define the probability of a single variable to be drawn in random sampling procedure. As a result, variables with low probabilities might not be drawn and are thus also removed from potential variable space. After a defined number of sampling runs the algorithm choses the overall best model.

In this study, PLSR modelling was divided into two blocks [block I and II, Fig. 1]. In the first block, we used random sampling to divide the 74 spectra into 44 spectra for model building (calibration data) and 30 spectra for an outer model validation [6]. We sampled randomly 100 times, saved these data splits and reused them in all following steps. To mirror the operational application of NIR spectroscopy for predicting fine-root lignin from unknown field samples, using an already established prediction model, calibration and prediction data sets were pre-processed separately [6]. To test the effects of different pre-processing procedures, we used raw data as well as six pre-processing methods: asymmetric least squares baseline offset correction (B.als)^83^, iterative restricted least squares baseline offset correction (B.irls)^84^, multiplicative scatter correction (MSC)^58^, standard normal variate (SNV)^85^, first derivative (D1) and second derivative (D2)^43^ [7]. Based on the differently pre-processed calibration data set the PLS regression models were computed and cross validated (ten-fold) to avoid model overfitting and to determine the optimal number of latent variables (nLV)^33^ [8]. Then all final models were applied to predict the respective prediction data [9]. From Block I we analyzed the obtained prediction accuracies averaged from 100 data splits and selected the pre-processing method with the highest accuracy for further analysis in Block II [10].

In the second block of model building, we used the CARS-PLSR approach as described above. We used the same data splits as in Block I to allow a direct comparison with the results of full spectrum-PLSR [10].

We describe model quality using the following indices: coefficient of determination for prediction (R_P_^2^)_,_ the root mean square error of cross validation (RMSECV), the root mean square error of prediction (RMSEP), the standard error of prediction (SEP) and the residual predictive deviation (RPD), calculated from standard deviation of the prediction data set and SEP. We consider RPD values >1.5–2 as good for preliminary screenings and initial predictions^86^, RPD values >2–2.5 as acceptable for quantitative predictions, values > 2.5–3 as good and values >3 as excellent for predictions^50^. We conducted all statistical analyses in the statistics software R (version 3.3.1)^87^ using the packages: baseline for B.als**/**B.irls corrections^84^, pls^88^, carspls^37^ (downloadable at: https://code.google.com/archive/p/carspls/downloads) and prospectr for SNV and MSC^89^.

## Supplementary information

S1

## Data Availability

The data used in this article is accessible via https://doi.pangaea.de/10.1594/PANGAEA.895501.
