# Supplementary material for: Fine root lignin content is well predictable with near-infrared spectroscopy
Source: Sci Rep. 2019 Apr 23;9:6396. doi: 10.1038/s41598-019-42837-z (PMC6479063; doi:10.1038/s41598-019-42837-z)
Supplement: Supplementary file 1 — S1 [file 41598_2019_42837_MOESM1_ESM.docx]

**Fine root lignin content is well predictable with near-infrared spectroscopy**

**Authors**

Oliver Elle^1,4 #^& Ronny Richter^1,2,3 #^, Michael Vohland^2,3^, Alexandra Weigelt^1,3^

**Affiliations**

# joined first authorship

^1^ Systematic Botany and Functional Biodiversity, Institute of Biology, Leipzig University, Johannisallee 21-23, D-04103 Leipzig, Germany

^2^ Geoinformatics and Remote Sensing, Institute for Geography, Leipzig University, Johannisallee 19a, D-04103 Leipzig, Germany;

^3^ German Centre for Integrative Biodiversity Research (iDiv) Halle-Jena-Leipzig, Deutscher Platz 5e, D-04103 Leipzig, Germany

^4^ Macromolecular Chemistry and Paper Chemistry, Ernst-Berl-Institute, Technische Universität Darmstadt, Alarich-Weiss-Straße 8, D-64287 Darmstadt, Germany

**Corresponding author**

Ronny Richter, German Centre for Integrative Biodiversity Research (iDiv) Halle-Jena-Leipzig, Deutscher Platz 5e, D-04103 Leipzig, Germany

Email: ronny.richter@idiv.de

**Supplement**

To put our results in a wider methodological and statistical perspective we performed a literature survey for studies predicting lignin content via spectroscopic measurements. We listed studies (1) measuring chemical content of lignin and using spectroscopy on plant tissue samples, (2) reporting the set of species used for lignin extraction, (3) using PLSR or other multivariate calibration methods to predict lignin contents spectrally and (4) validating the model on independent test data. We identified 30 relevant studies, which predicted lignin content via spectroscopic measurement following our four prerequisites (see supplementary table S1 for full details of individual studies).

**Table S1:** Overview of detail information of all studies included in our literature survey. Presented are the material of interest (Material), the number of species included in the study (Species), type of lignin extractives (Lignin) with the categories (AIR = acid-insoluble residue, determined with Klason method; ADL = acid detergent lignin, determined with van Soest method; AIL = acid-insoluble lignin, determined with Klason method; ASL = acid-soluble lignin, determined with Klason method), sample mass for lignin extraction (Mass), mean lignin content and the standard deviation in the overall sample set (Mean_T_, SD_T_) and mean lignin content and the standard deviation in the sample set used for validation (Mean_v_, SD_v_), number of samples (Samples), the ratio of samples used for model validation and calibration (Val/Cal), root mean square error of prediction (RMSEP), standard error of prediction (SEP), measure of determination between predicted and observed lignin contents (R_p_^2^) and residual predictive deviation (RPD) as reported in single studies; (/) indicates that value was not reported.

| **Nr.** | **Study** | **Material** | **Species** | **Lignin** | **Mass** | **Mean_T_** | **SD_T_** | **Mean_V_** | **SD_V_** | **Samples** | **Val / Cal** | **RMSEP** | **SEP** | **R_p_^2^** | **RPD** | |
| --- | --- | --- | --- | --- | --- | --- | --- | --- | --- | --- | --- | --- | --- | --- | --- | --- |
|  |  |  | **[N]** | **[Method]** | **[mg]** | **[%]** | **[%]** | **[%]** | **[%]** | **[N]** |  | **[%]** | **[%]** |  |  | |
| 1 | Assis (2017) | bagasse | 1 | AIR | 300 | 23.44 | 0.96 | / | / | 378 | 0.12 | 0.85 | / | 0.94 | 2.87 | |
| 2 | Baloyi (2013) | herb, grass | 5 | ADL | 500 | / | / | 10.90 | 3.36 | 79 | 1.47 | / | 2.96 | / | 1.14* | |
| 3 | Castillo (2014) | wood | 3 | AIR + ASL | 300 | / | / | 22.20 | 8.60 | 36 | 0.38 | 2.10 | / | 0.94 | 4.10 | |
| 4 | Fahey (2018)^3^ | wood | 1 | AIR | 100 | / | / | / | / | 17 | 0.55 | 2.51 | 2.27 | / | / | |
| 4 | Fahey (2018)^4^ | wood | 1 | AIR | 100 | / | / | / | / | 50 | 3.55 | 3.43 | 3.33 | / | / | |
| 5 | Guimarães (2014) | stalks | 1 | ADL | 500 | / | / | / | / | 788 | 0.48 | 0.80 | / | / | 2.00 | |
| 6 | Hayes(2015) | peat | 1 | AIL | / | 57.27 | 7.61 | / | / | 53 | 0.33 | 2.03 | 2.10 | 0.94 | 3.49 | |
| 7 | Huang (2008) | wood | 1 | AIR | 1000 | 23.87 | 2.46 | / | / | 53 | 0.50 | / | 0.65 | 0.94 | 3.78 | |
| 7 | Huang (2008) | wood | 1 | AIR | 1000 | 34.11 | 0.64 | / | / | 46 | 0.50 | / | 0.28 | 0.81 | 2.29 | |
| 7 | Huang (2008) | wood | 1 | AIR | 1000 | 18.11 | 1.81 | / | / | 64 | 0.50 | / | 0.61 | 0.74 | 2.97 | |
| 7 | Huang (2008) | wood | 1 | AIR | 1000 | 18.24 | 1.07 | / | / | 50 | 0.50 | / | 0.81 | 0.45 | 1.32 | |
| 8 | Jiang (2014) | wood | 1 | AIR | 300 | / | / | 26.63 | 1.05 | 21 | 0.31 | 0.19 | / | 0.99 | 5.53 | |
| 9 | Jin (2017) | stalks | 1 | ADL | 500 | / | / | / | / | 179 | 0.49 | / | 0.55 | 0.86 | 2.32 | |
| 10 | Jones (2008) | wood | 1 | AIR | / | 29.97 | 0.82 | / | / | / | / | / | / | 0.73 | 1.57 | |
| 11 | Karlinasari (2014) | wood | 1 | AIL + ASL | 1000 | / | / | / | / | 399 | 0.48 | / | 2.04 | 0.18 | 1.07 | |
| 12 | Kelley (2004) | fiber | 14 | AIL | 100 | / | / | / | / | 23 | / | / | / | 0.50 | / | |
| 13 | Kong (2005) | stalks | 2 | ADL | 500 | / | / | 6.95 | 1.57 | 203 | 0.50 | / | 0.65 | 0.83 | 2.43 | |
| 14 | Lavin (2015) | leaf | 1 | ADL | 500 | / | / | 15.70 | / | 164 | 0.14 | / | 4.28 | 0.52 | / | |
| 14 | Lavin (2015) | wood | 1 | ADL | 500 | / | / | 13.80 | / | 164 | 0.14 | / | 3.71 | 0.33 | / | |
| 15 | Li (2015) | stalks | 1 | ADL | 500 | 20.35 | 1.84 | 20.36 | 1.84 | 171 | 0.50 | / | 0.60 | 0.89 | 3.13 | |
| 16 | Louhelainen (2017) | wood | 1 | AIL + ASL | 200 | / | / | / | / | 48 | 0.23 | 1 | / | 0.90 | / | |
| 16 | Louhelainen (2017) | wood | 1 | AIL + ASL | 200 | / | / | / | / | 48 | 0.23 | 0.50 | / | 0.88 | / | |
| 17 | McLean (2014) | wood | 1 | AIR | 200 | / | / | 32.51 | 5.10 | 147 | 2.13 | 1.03 | / | 0.95 | 4.95* | |
| 18 | Monrroy (2015) | wood | 2 | AIR + ASL | 300 | 20.10 | 0.30 | / | / | 89 | 0.27 | 0.90 | 0.90 | 0.99 | 9.50 | |
| 19 | Niu (2014) | straw | 1 | AIL + ASL | 300 | 20.22 | 2.60 | / | / | 109 | 0.49 | 1.47 | / | 0.65 | 1.76 | |
| 20 | Ono (2003) | various^a^ | 32 | AIR + ASL | 200 | 31.10 | 10.1 | / | / | 129 | 0.52 | / | 5.00 | 0.76 | 2.10 | |
| 21 | Petisco (2006) | leaf | 17 | ADL | 500 | / | / | 14.70 | 4.17 | 182 | 0.64 | / | 0.85 | 0.95 | 4.39* | |
| 22 | Rambo (2015) | residues | 1 | AIL + ASL | 300 | 24.80 | 4.31 | / | / | 27 | 0.35 | 1.74 | / | 0.83 | / | |
| 23 | Rijal (2016)^1^ | leaf | 1 | AIL + ASL | 300 | / | / | 17.03 | 1.73 | 152 | 0.49 | 3.48 | / | 0.05 | 0.50* | |
| 23 | Rijal (2016)^2^ | leaf | 1 | AIL + ASL | 300 | / | / | 18.02 | 1.62 | 102 | 0.31 | 1.74 | / | 0.36 | 0.93* | |
| 24 | Xue (2015) | stover | 1 | AIR + ASL | 300 | / | / | 23.18 | 1.90 | 217 | 0.33 | 1.17 | / | 0.61 | 1.62* | |
| 25 | Yao (2010) | wood | 5 | AIR + ASL | 1000 | / | / | 21.66 | 1.33 | 78 | 0.30 | 0.53 | / | 0.88 | 2.53* | |
| 26 | Ye (2008) | various^b^ | 1 | AIR + ASL | 300 | 22.10 | 2.25 | / | / | 35 | 0.17 | / | 1.12 | 0.88 | / | |
| 27 | Yeh (2004) | wood | 1 | AIR + ASL | 1000 | / | / | / | / | / | / | / | 0.87 | 0.72 | / | |
| 28 | Yeh (2005) | wood | 1 | AIR + ASL | 100 | / | / | 29.80 | 0.90 | 53 | 0.36 | / | 0.63 | 0.52 | 1.49 | |
| 28 | Yeh (2005) | wood | 1 | AIR + ASL | 100 | / | / | 24.60 | 1.90 | 53 | 0.36 | / | 0.74 | 0.89 | 2.58 | |
| 29 | Zhou (2015) | wood | 4 | AIR | 2500 | 26.24 | 2.41 | / | / | 37 | 0.16 | 0.46 | / | / | 5.24 | |
| 30 | Zhou (2016) | wood | 1 | AIR + ASL | 500 | / | / | / | / | 70 | 0.17 | 0.56 | / | 0.87 | 2.47 | |
|  | | | | | | | | | | | | | | | |  |
| 1) across year, 2) across cultivars, 3) glasshouse, 4) semi-natural | | | | | | | | | | | | | | | |  |
| a) leaf, litter fall, organic material, b) cornstover fractions (leaf, pith, node, rind, sheath, husk) | | | | | | | | | | | | | | | |  |
| * indicates RPD calculated from SD_V_ and RMSEP | | | | | | | | | | | | | | | |  |
|  | | | | | | | | | | | | | | | |  |
